# Pathway Preferential Estrogens Prevent Hepatosteatosis Due to Ovariectomy and High-Fat Diets

**DOI:** 10.3390/nu13103334

**Published:** 2021-09-23

**Authors:** Qianying Zuo, Karen L. Chen, Alicia Arredondo Eve, Yu-Jeh Liu, Sung Hoon Kim, Benita S. Katzenellenbogen, John A. Katzenellenbogen, Zeynep Madak-Erdogan

**Affiliations:** 1 Department of Food Science and Human Nutrition, University of Illinois at Urbana-Champaign, Urbana, IL 61801, USA; qzuo2@illinois.edu (Q.Z.); aliciaa2@illinois.edu (A.A.E.); jayliu1117@gmail.com (Y.-J.L.); 2 Division of Nutritional Sciences, University of Illinois at Urbana-Champaign, Urbana, IL 61801, USA; kchen9282@gmail.com; 3 Department of Chemistry, University of Illinois at Urbana-Champaign, Urbana, IL 61801, USA; kimsh@illinois.edu (S.H.K.); jkatzene@illinois.edu (J.A.K.); 4 Department of Molecular and Integrative Physiology, University of Illinois at Urbana-Champaign, Urbana, IL 61801, USA; katzenel@illinois.edu; 5 Cancer Center at Illinois, University of Illinois at Urbana-Champaign, Urbana, IL 61801, USA; 6 Beckman Institute of Technology, University of Illinois at Urbana-Champaign, Urbana, IL 61801, USA; 7 Institute for Genomic Biology, University of Illinois at Urbana-Champaign, Urbana, IL 61801, USA

**Keywords:** non-alcoholic fatty liver disease (NAFLD), hepatosteatosis, pathway preferential estrogen 1 (PaPE-1), high-fat diet, metabolic health

## Abstract

About 20–30% of premenopausal women have metabolic syndrome, and the number is almost double in postmenopausal women, and these women have an increased risk of hepatosteatosis. Postmenopausal women with metabolic syndrome are often treated with hormone replacement therapy (HRT), but estrogens in currently available HRTs increase the risk of breast and endometrial cancers and Cardiovascular Disease. Therefore, there is a critical need to find safer alternatives to HRT to improve postmenopausal metabolic health. Pathway preferential estrogen 1 (PaPE-1) is a novel estrogen receptor ligand that has been shown to favorably affect metabolic tissues without adverse effects on reproductive tissues. In this study, we have examined the effects of PaPE-1 on metabolic health, in particular, examining its effects on the liver transcriptome and on plasma metabolites in two different mouse models: diet-induced obesity (DIO) and leptin-deficient (ob/ob) mice. PaPE-1 significantly decreased liver weight and lipid accumulation in both DIO and ob/ob models and lowered the expression of genes associated with fatty acid metabolism and collagen deposition. In addition, PaPE-1 significantly increased the expression of mitochondrial genes, particularly ones associated with the electron transport chain, suggesting an increase in energy expenditure. Integrated pathway analysis using transcriptomics and metabolomics data showed that PaPE-1 treatment lowered inflammation, collagen deposition, and pathways regulating fatty acid metabolism and increased metabolites associated with glutathione metabolism. Overall, our findings support a beneficial metabolic role for PaPE-1 and suggest that PaPE-1 may protect postmenopausal women from fatty liver disease without increasing reproductive cancer risk.

## 1. Introduction

Non-alcoholic fatty liver disease (NAFLD) is the most common form of chronic liver disease in the United States [1]. Simple steatosis is considered benign, but steatosis of the liver can progress to the more serious conditions of nonalcoholic steatohepatitis (NASH) and cirrhosis [2,3]. The “2-hit hypothesis” regarding NAFLD pathogenesis suggests that NAFLD is caused by two major factors: the accumulation of excessive hepatic fat and the onset of oxidative stress [4,5,6]. Therefore, a treatment that targets one or ideally both of these aspects may be beneficial in mitigating NAFLD and the risk of other ensuing morbidities.

Population studies have demonstrated that men and postmenopausal women have higher incidences of NAFLD compared to premenopausal women, suggesting a protective role for estrogens [7,8]. The decrease in estrogens due to the onset of menopause makes postmenopausal women more susceptible to weight gain, fat redistribution to abdominal areas, dyslipidemia, hypertension, and insulin resistance, all of which are major hallmarks of metabolic syndrome and are associated with NAFLD [9]. 

These effects, including insulin resistance and glucose homeostasis, can be reversed by estradiol (E2) treatment [10,11]. Current hormone replacement therapy (HRT) typically consists of estrogen or estrogen in combination with a progestin and improves postmenopausal symptoms. However, concerns have been raised about the side effects of HRTs, such as the increased risk of breast and endometrial cancers, deep vein thrombosis, and stroke. Therefore, it is essential to find safer alternatives to HRT that can maintain the health of non-reproductive tissues without stimulating reproductive tissues. 

We have previously developed novel pathway preferential estrogens that modulate extranuclear-initiated estrogen receptor α (ERα) and mTOR signaling crosstalk [12]. The lead compound, termed Pathway Preferential Estrogen 1 (PaPE-1), is structurally similar to E2. Because of the rearrangement of the key elements of the steroid structure, PaPE-1 has a lower binding affinity for ERα. Thus, PaPE-1 selectively activates extranuclear-initiated ERα signaling pathways without triggering gene regulation of classical ER target genes. In in vivo studies, PaPE-1 reduced ovariectomy-induced weight gain, blood triglyceride levels, and fat deposition, without stimulating the uterus or mammary gland. In another previous study, PaPE-1 also effectively blocked gene expression changes in breast cancer induced by free fatty acids, providing a mechanistic rationale for clinically evaluating PaPE-1 for decreasing breast cancer progression in obese postmenopausal women [13].

In this study, we analyze the metabolic effects of PaPE-1, characterize the signaling pathways and markers of NAFLD that it modulates, and evaluate the potential of PaPE-1 to combat hepatic steatosis caused by decreased estrogens. To understand the molecular mechanism and systemic effects of PaPE-1 on metabolism in the low-estrogen environment, we performed genomic and postgenomic analyses in female mouse models of diet-induced and genetic obesity in a low-estrogen background. Our analysis reveals a significant and potentially beneficial role for PaPE-1 in modulating the expression of genes that regulate lipid deposition, mitochondrial activity, and collagen accumulation. Our combined liver gene-expression and blood metabolomics analyses show that PaPE-1 is effective in normalizing fatty acid metabolism and inflammatory pathways associated with the liver. Our findings in experimental mouse models suggest a potentially beneficial role for PaPE-1 in protecting postmenopausal women from NAFLD without increasing reproductive cancer risk.

## 2. Materials and Methods

### 2.1. Animal Models and Treatments 

Studies used wild-type and Leptin mutant (ob/ob) mice in the C57BL/6 background. All experiments involving animals were conducted in accordance with National Institutes of Health standards for the use and care of animals, with protocols approved by the University of Illinois at Urbana-Champaign (IACUC protocol #14193). Based on our previous work on body weight normalization by low-affinity estrogens in mice treated with controls or various estrogens, typical treatment differences in the weight of animals on a high-fat diet following ovariectomy is 5 g; standard deviations were approximately 3 g. Using these predictions and a Type I error of 5% and a Type II error of 10%, we estimated that 8 animals were required for each group in each experiment [12,14]. 

Two different animal models for obesity were utilized for this study. The first model is the diet-induced obesity (DIO) model, using 24 female wild-type C57BL/6 (Jackson Laboratory) mice housed individually in 12-h light-dark cycle. Mice were given a high-fat diet (Harlan TD 0.88137), based on an AIN-76A diet but with 45% calories from fat and 0.2% cholesterol. After one week, all mice were ovariectomized (OVX) under isoflurane anesthesia and treatment was administered through a 60-day release osmotic minipump (Alzet 2006, DURECT Corporation; flow rate 0.15 µL/h). The second model is a genetic model of obesity, using 24 female leptin-deficient (ob/ob) mice from a C57BL/J6 background (RRID: IMSR_JAX:000664, Jackson Laboratory) housed individually in 12-h light-dark cycle and given a normal AIN-76A diet. One week after ovariectomy, mice were randomized into different treatment groups. Treatment groups for both models were the same: (1) vehicle of 43% DMSO, 15% ethanol, and 42% saline; (2) E2 at 5 µg·kg^−1^·day^−1^; (3) PaPE-1 at 300 µg·kg^−1^·day^−1^. Treatments were administered through a 60-day release osmotic minipump (Alzet 2006, DURECT Corporation; flow rate 0.15 µL/h) for 6 weeks. All mice were given water ad libitum.

### 2.2. Food Intake and Body Composition

Food intake and body weight were measured weekly for six weeks. MRIs were performed before treatment. At week 4, after treatment started, EchoMRI was used to measure the whole body, lean, and water mass. After six weeks of treatment, mice were euthanized, and organs were harvested. The liver and uterus were weighed and recorded. Livers were kept in RNAlater (Thermo Scientific, Waltham, MA, USA) for RNA isolation or 10% neutral buffered formalin (Millipore Sigma, Burlington, MA, USA) for histology and staining. 

### 2.3. Liver Histology

Liver tissue sections were embedded in the Tissue-Tek O.C.T. compound and frozen. Sections were fixed in 10% neutral buffered formalin (NBF) then stained with hematoxylin and eosin (H&E) or Oil Red O. Paraffin-wax-embedded liver sections were also stained with Trichrome and Sirius Red. Stained tissue sections were imaged using EVOS XL Core Imaging System. Lipid droplet size was quantified on H&E stains using particle analysis on Fiji software (ImageJ). Lipid droplet count was quantified by manually counting on Fiji. 

### 2.4. RNA-Seq and Transcriptional Profiling

Total RNA was extracted with TRIzol reagent (Life Technologies, Carlsbad, CA, USA) according to the manufacturer’s protocol and was purified using an RNAeasy kit (QIAGEN, Hilden, Germany). RNA quality was assessed using BioTek Cytation 5. Concentrated RNA was submitted to the DNA Sequencing Group at the Roy J. Carver Biotechnology Center at UIUC. Complementary DNA (cDNA) libraries were prepared with the mRNA TruSeq Kit (Illumina Inc., San Diego, CA, USA). Double-stranded cDNA was generated from fragmented RNA, and adapters were ligated to the ends. Casava 1.8.2. was used to base call and demultiplex samples. Preprocessing and Quality Control: FASTQ files were trimmed using FASTQ Trimmer (version 1.0.0). TopHat (version 0.5) was used to map single-end RNA-seq reads to the Mus musculus reference genome. Gene expression values were quantified from BAM files, calculated using StrandNGS (version 2.1) Quantification tool. Partial reads were considered, and the option for detecting novel genes and exons was selected. Default parameters for finding novel exons and genes were specified. Quality control and normalization were conducted in R using edgeR (Version 3.24.3). Statistical analysis was conducted in R using limma (Version 3.38.3). DESeq normalization algorithm using default values was chosen. Differentially expressed genes were then determined by fold-change and *p*-value with Benjamini and Hochberg multiple test correction for each gene for each treatment relative to the vehicle control. We considered genes with fold-change > 2 and FDR (or q) < 0.05 as being significantly, differentially expressed. Gene Set Enrichment Analysis (GSEA) was used to identify GO terms associated with different treatments [15,16].

### 2.5. Cell Culture, Ligand Treatments, Reagents

HepG2-ERα cells were obtained from Dr. David Shapiro (University of Illinois) and grown as previously described [14]. Cells were maintained in William’s medium (Gibco, Waltham, MA, USA) with phenol red and non-essential amino acid salts (NEAA), supplemented with 5% serum + II (HyClone, Logan, UT, USA), 100 µg/mL penicillin/streptomycin (Invitrogen, Carlsbad, CA, USA), and 2.5% sodium bicarbonate. Cells were grown for at least 5 days in this media prior to use. William’s medium without phenol red was used for treating HepG2-ERα cells. Oleic acid (OA) used in the cell assays was purchased from Sigma Aldrich and it was dissolved in a small amount of DMSO and brought to the desired concentration by adding ethanol. 

### 2.6. Real-Time RT-qPCR

HepG2-ERα Cells were seeded on 30 mm cell culture plates at 5 × 105 cells/well in William’s medium without phenol red and were treated with medium containing with 1 μM PaPE-1 or 0.01 μM Estradiol for 24 h. The next day, HepG2-ERα cells were homogenized in 1 mL of TRIzol reagent, and total RNA was isolated. RNA of liver tissues and HepG2-ERα Cells was transcribed into cDNA using M-MuLV Reverse Transcriptase (BioLabs, Ipswich, MA, USA). Fast Start Universal SYBR Green reagent [17] and an Applied Biosystem Step One Plus qPCR system were used for the RT-qPCR reaction. Results were normalized to housekeeping gene 36B4 (60S acidic ribosomal protein P0), and the relative difference in gene expression from the vehicle was calculated using the ∆∆Ct method. PCR primer sequences were obtained from PrimerBank (https://pga.mgh.harvard.edu/primerbank/, accessed on 20 September 2017): 36B4, FASN (Fatty Acid Synthase), SREBP-1c (Sterol regulatory element-binding protein 1), COL4A1 (Collagen alpha-1 (IV) chain), COL5A3 (Collagen alpha-3 (V) chain), COL15A1 (Collagen alpha-1 (XV) chain), FDX1 (Adrenal ferredoxin), ETFB (Electron Transfer Flavoprotein Subunit Beta), NDUFB4 (NADH: Ubiquinone Oxidoreductase Subunit B4) and NDUFA2 (NADH: Ubiquinone Oxidoreductase Subunit A2).

### 2.7. Seahorse Metabolic Profiling Assays

HepG2-ERα cells were seeded at a density of 5 × 10^4^ in William’s medium (Gibco, Waltham, MA, USA) with phenol red. The next day, HepG2-ERα cells were treated in triplicate using Veh, 1 μM PaPE-1, 100 nM OA with/without 1 μM PaPE-1 in corresponding treatment media without phenol red in each well of the XFp Cell Culture miniplates, respectively (Seahorse Bioscience Inc., Billerica, MA, USA) for 24 h. Concentrations of ligands are based on our previously published study (12). The cartridges were hydrated with the calibration solution and kept in a non-CO_2_ incubator at 37 °C overnight. In parallel, a duplicate of each plate was used for cell counting to monitor cell number changes after 24 h of treatments, and Seahorse data was normalized to total cell number. On the assay day, cells were washed with XF Base Media without phenol red supplemented with 10 mM L-glucose, 2 mM L-glutamine (Gibco, Waltham, MA, USA), and 1 mM sodium pyruvate (Gibco, Waltham, MA, USA) and incubated in the same media for 45–60 min before assays were run with Seahorse XFp Analyzer (Seahorse Bioscience Inc., North Billerica, MA, USA). Mitochondrial energy production was measured using the Seahorse XFp Cell Mito Stress Test Kit (Seahorse Bioscience Inc., North Billerica, MA, USA). Experiments were performed in triplicate and repeated at least three times.

### 2.8. Neutral Lipid Droplet Staining

HepG2-ERα cells seeded in 96-well plates were fixed with 4% formaldehyde in 10% phosphate-buffered saline (PBS) (Millipore Sigma, Burlington, MA, USA). After fixation, cells were washed with 10% PBS and stained using Invitrogen’s HCS LipidTox Neutral Green Lipid Stain for cellular imaging. Cells were counterstained with DAPI (Millipore Sigma, Burlington, MA, USA) and viewed via BioTek Cytation 5 according to the manufacturer’s recommendations. Corrected total cell fluorescence (CTCF) was calculated using Fiji software by taking the integrated density, area, and mean fluorescence of background and performing the calculation: CTCF = Integrated Density- (Area of Selected Cell x Mean Fluorescence of Background).

### 2.9. Metabolomics Analysis

About 500 µL of blood was collected from the mouse abdominal aorta. Blood collected at the end of the experiment was mixed with 10 µL of 0.5 M EDTA and centrifuged for 10 min at 1000× *g* to isolate the plasma. Plasma samples from each animal were collected and stored at -80 °C until submitted to the Metabolomics Center in the Roy J Carver Biotechnology Center (CBC) at UIUC. GC/MS whole metabolite profiling was performed to detect and quantify the metabolites by using Gas chromatography-mass spectrometry (GC/MS) analysis. Metabolites were extracted from 50 µL of plasma according to Agilent Inc. application notes. The hentriacontanoic acid was added to each sample as the internal standard prior to derivatization. Metabolite profiles were acquired using an Agilent GC-MS system (Agilent 7890 gas chromatograph, an Agilent 5975 MSD, and an HP 7683B autosampler). The spectra of all chromatogram peaks were evaluated using the AMDIS 2.71 and a custom-built database with 460 unique metabolites. All known artificial peaks were identified and removed prior to the data mining. To allow comparison between samples, all data were normalized to the internal standard in each chromatogram. This analysis identifies about 150 plasma metabolites and reports a relative abundance of metabolites, which enables us to compare the same metabolites across the sample batch. Metabolomics data with sample class annotations (Vehicle, E2, and PaPE-1) were uploaded to the Statistical Analysis tool of MetaboAnalyst software version 4.0 [15]. Features with more than 50% missing values were removed. Data were normalized based on values from Vehicle samples. Data were log-transformed and scaled using the Auto scaling feature. Heatmap of class averages of 25 metabolites was generated using the Heatmap feature using default options for clustering and restricting the data to the top 25 metabolites ranked by *t*-test. PLS Discriminant Analysis (PLS-DA) is performed in order to sharpen the separation between Vehicle, E2, and PaPE-1 groups to show distinct metabolic profiling. VIP scores for the top 25 metabolites that discriminate between treatment groups were calculated and displayed using the Partial Least Squares-Discriminant analysis tool. Fold change analysis was performed to compare the absolute value of change of metabolites between two group means. Enrichment analysis and pathway analysis were used to identify metabolic pathways associated with enriched metabolites (fold change > 2 and *p* < 0.05).

### 2.10. Statistical Analysis

Data from gene expression and animal studies were analyzed using either a one-way analysis of variance (ANOVA) model to compare different ligand effects, or two-way-ANOVA to compare time-dependent changes. Statistical significance was established at α = 0.05. All data was normally distributed unless otherwise noted. Normally distributed data was analyzed using pairwise *t*-tests with a Bonferroni correction to identify treatments that were significantly different from each other (* *p* < 0.05, ** *p* < 0.01, **** *p* < 0.0001). For every main effect that was statistically significant at α = 0.05, pairwise *t*-tests were conducted to determine which ligand or inhibitor treatment levels were significantly different from each other. For these *t*-tests, the Bonferroni correction was employed to control experiment-wise type I error rate at α = 0.05 followed by the Bonferroni post hoc test. Data that were not normally distributed were analyzed using Mann Whitney test for nonparametric data (* *p* < 0.05, ** *p* < 0.01, **** *p* < 0.0001). Statistical significance was calculated using GraphPad Prism for Windows (GraphPad Software, La Jolla, CA, USA, www.graphpad.com, accessed on 20 September 2017). The mean and SEM of experiments were plotted.

## 3. Results

### 3.1. PaPE-1 Was Effective in Decreasing Ovariectomy-Associated Weight Gain and Hepatic Lipid Deposition without Stimulating Reproductive Tissues

**Figure 1:**
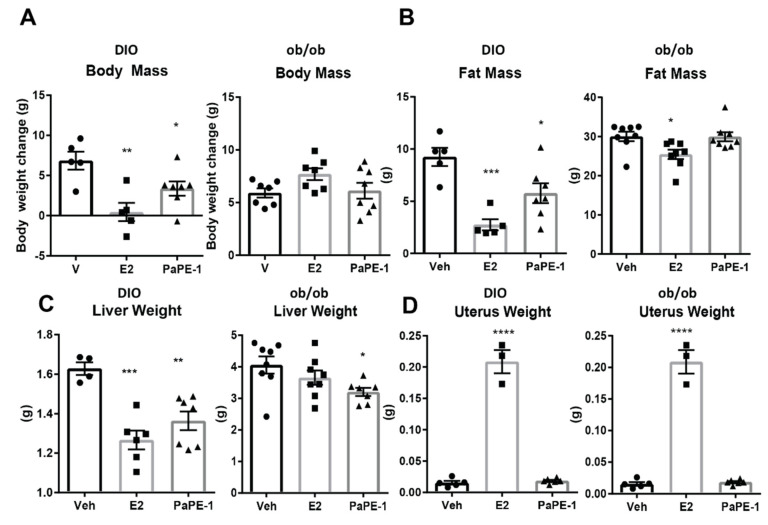
Body and organ mass of E2 and PaPE-1 treated diet induced obesity (DIO) and ob/ob mice. (**A**) PaPE-1 decreased weight gain caused by high-fat diet (HFD) in DIO mice, but not significantly in ob/ob mice. (**B**) E2 and PaPE-1 prevent fat mass increase caused by HFD but not in ob/ob mice. (**C**) The liver weight of both DIO and ob/ob mice showed that PaPE-1 was very effective at decreasing liver weight increase caused by HFD in DIO mice or leptin deficiency. (**D**) Unlike E2 treatment, PaPE-1 does not increase uterus weight in both DIO and ob/ob mice. Statistical significance was established at α = 0.05. All weights were normally distributed. Pairwise *t*-tests with a Bonferroni correction were used to identify treatments that were significantly different from each other (* *p* < 0.05, ** *p* < 0.01, *** *p* < 0.001, **** *p* < 0.0001).

Body weights of wild-type high-fat-fed or diet-induced obesity (DIO) mice were significantly lowered by E2 and PaPE-1 after six weeks of treatment, as we noted previously (Figure 1A) [12]. In ob/ob mice, PaPE-1 did not significantly change body weight compared to the control, whereas E2 treatment increased body weight (Figure 1A) [12]. As shown in the MRI analyses, however, the increase in body weight in E2-treated animals was not attributed to fat mass, but rather to the water and lean mass of the animals. MRI analysis showed that E2 and PaPE-1 significantly decreased fat mass in DIO mice, whereas only E2 decreased fat mass in ob/ob mice (Figure 1B). At harvest after six weeks of treatment, liver weights of E2 and PaPE-1-treated animals were significantly lower than the vehicle in both DIO mice and ob/ob models, while PaPE-1 was more effective in the ob/ob model (Figure 1C). Furthermore, as expected, PaPE-1 treatment, unlike E2 treatment, did not increase uterine weight in both models (Figure 1D).

**Figure 2:**
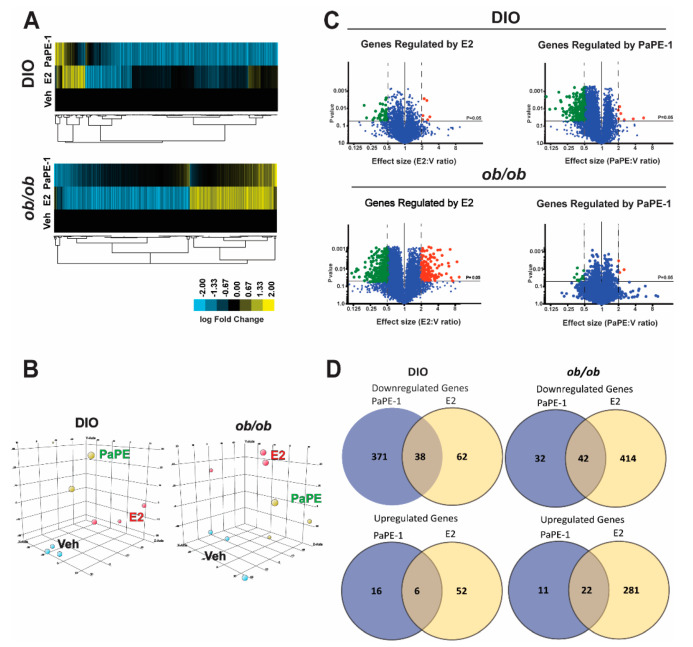
Transcriptomic changes in the liver from DIO and ob/ob mice. (**A**) RNA was isolated from the liver and RNA-seq was performed. Regulated genes are those with *p* < 0.05 and expression fold change > 2 (*n* = 3 biological replicates). (**B**) PaPE-1and E2 treatments show distinct gene regulation patterns. (**C**) Volcano plots of gene expression data show several genes significantly affected by PaPE-1 treatment differ from those affected by E2 treatment. Statistical significance was determined at *p* = 0.05 and greater than a 2-fold change. Significantly downregulated genes are highlighted in green and upregulated genes are highlighted in red. (**D**) The Venn diagrams display the relationships of downregulated and upregulated genes between DIO and ob/ob mice, respectively. The genes shown in the diagrams are all genes significantly regulated by E2 and PaPE-1.

As the reduction of liver weight was common in both models, we focused on the liver phenotype. To assess the changes in gene expression programs modulated by estrogen deficiency and high-fat diets, and normalized by E2 and/or PaPE-1 supplementation, RNA was extracted from the mouse liver and RNA-seq was performed. Regulated genes are considered to be those with expression fold change >2 at *p*-value < 0.05 (*n* = 3 biological replicates). Based on transcriptional profiling of these treatments, PaPE-1 up-regulates and down-regulates common as well as distinct groups of genes compared to those regulated by E2 (Figure 2A,B,D). Volcano plots of gene expression data show several genes that were significantly affected by PaPE-1 treatment differently than from E2 treatment. Significantly downregulated genes are highlighted in green and upregulated genes are highlighted in red (Figure 2C). Statistical significance was determined at α = 0.05. Each row was analyzed individually, without assuming a consistent standard deviation. Highly significant regulated genes were considered to be those having a statistical significance of *p* < 0.05 and a greater than 2-fold change compared to vehicles. Several genes considered highly significant were downregulated and upregulated by PaPE-1 treatment. These genes were used for subsequent integrated analyses (Supplemental data S1).

### 3.2. PaPE-1 Alleviated Hepatic Steatosis in Obese Mice and Decreased Lipid Accumulation in Hepg2-ERα Cells

**Figure 3:**
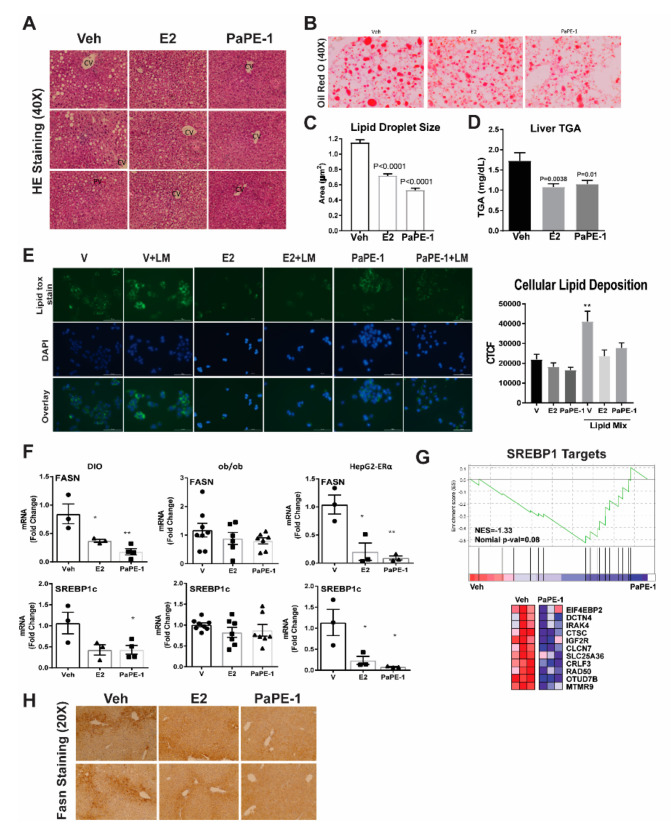
Lipid accumulation in mouse liver and human hepatocyte cells(HepG2-ERα) after E2 and PaPE-1 treatments (**A**,**B**) H + E and Oil Red O staining of mice liver tissue show decreased lipid accumulation after both E2 and PaPE1 treatments. CV, centrilobular vein; PV, periportal vein. (**C**) Quantification of lipid droplet sizes by Fiji software correlates to show PaPE1 is effective at decreasing lipid droplet size in mice liver. (**D**) Liver triglyceride (TGA) level decreased after both E2 and PaPE1 treatments (**E**) Lipid tox stain shows the lipid accumulation in different treatments. Cells were counterstained with DAPI via BioTek Cytation 5. Corrected total cell fluorescence (CTCF) shows the cellular lipid deposition, which was calculated using Fiji software. (**F**) PaPE-1 reduces the expression of FASN and SREBP-1c mRNA in both animal models and HepG2-ERα cells. (**G**) Consistent with a decrease in SREBP1 level, PaPE-1 reduced the expression of SREBP1 targets. (**H**) Expression of Fasn protein is also decreased in livers from animals treated with E2 or PaPE-1. * *p* < 0.05; ** *p* < 0.01.

To determine if the liver weight difference was due to lipid, histology analysis was performed. PaPE-1 and E2-treated animals had a significant reduction in hepatic steatosis compared to vehicle-treated animals (Figure 3A). The decreased steatosis visualized by Oil Red O staining correlated with the decreased liver weights in these animals, suggesting that the decreased liver weights were due to decreased steatosis and lipid deposition (Figure 3B). Quantification of lipid droplet size based on Oil Red O staining and comparing liver triglyceride levels in mice from different treatment groups confirmed a significant decrease in lipid deposition in both estrogenic treatments (Figure 3C,D). We verified the effects of PaPE-1 and E2 in human hepatocarcinoma HepG2-ERα cells by treating with the vehicle, E2, and PaPE-1, with and without the addition of lipids (Lipid Mix, LM) to the media to mimic blood composition in obese individuals. Then, we visualized the lipid accumulation using fluorescent LipidTox Neutral Green Lipid Stain (Figure 3E). Quantification was done using Fiji software. E2 and PaPE-1 were both effective in reducing lipid accumulation in HepG2-ERα cells in the presence of LM, which provided a quantitative assay to monitor estrogen regulation of lipid accumulation in liver cells. 

qPCR validation of selected genes FASN and SREBP-1c showed correlation with RNA-seq data (Figure 3F). PaPE-1 significantly decreased FASN and SREBP-1c gene expression in ovariectomized mice fed a high-fat diet. In ob/ob mice, suppression of FASN and SREBP-1c expression level was not statistically significant, but the trend was still present. To confirm this in hepatocyte cells, HepG2-ERα cells were treated with vehicle, E2, and PaPE-1 in the presence of LM and qPCR was performed (Figure 3F). Consistent with a decrease in SREBP1 level, PaPE-1 reduced the expression of SREBP1 targets (Figure 3G). With hormone treatment, FASN and SREBP-1c gene expression were both significantly decreased in HepG2-ERα cells as they were in the two mouse models. Fasn protein expression was also downregulated in livers from animals that received E2 or PaPE-1 treatments (Figure 3H). 

### 3.3. PaPE-1 Increases Overall Mitochondrial Protein Expression and Activity in Liver

**Figure 4:**
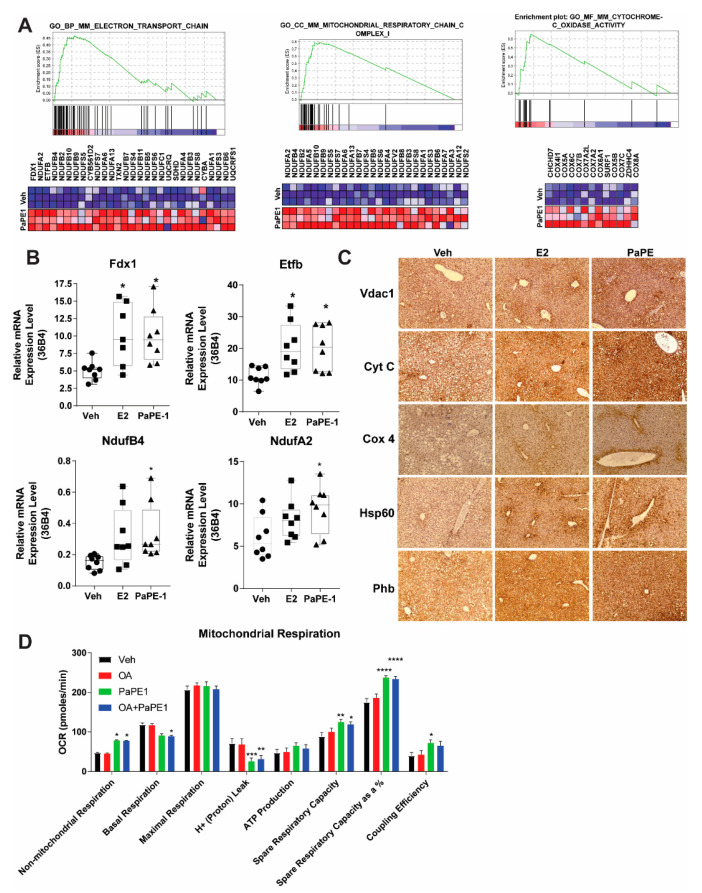
Increased mitochondrial gene and protein expression in PaPE-1 treated livers. (**A**) Examples of GSEA metabolic pathways. Electron transport chain, Mitochondrial respiratory chain, and cytochrome oxidase activity were three of the highly enriched gene sets in GSEA analysis. Range of colors (red, pink, light blue, dark blue) corresponds to a range of expression values (high, moderate, low, lowest). (**B**) PaPE-1 mediated the upregulation of the genes in these gene sets. (**C**) Consistent with our GSEA results, PaPE1 treated livers had an overall higher expression of mitochondrial proteins. (**D**) HepG2-ERα cells were treated in triplicate using Veh, 1 μM PaPE-1, 100 nM OA, and 1 μM PaPE-1 + 100 nM OA. Different oxidative respiration parameters were calculated using a Seahorse Mitostress test kit. *, *p* < 0.05; **, *p* < 0.01; ***, *p* < 0.001; ****, *p* < 0.0001.

Gene expression analysis pointed out a potential change in metabolic pathways in high-fat-fed mouse liver upon PaPE-1 exposure. GSEA analysis of liver samples showed that after PaPE-1 treatment, metabolic pathways involving electron transport chain, mitochondrial respiratory chain, and cytochrome oxidase activity were overall increased (Figure 4A). qPCR assays were performed with the RNA extracted from the liver samples from animals treated with Veh, E2, or PaPE-1, and general mitochondrial metabolism was increased, as reflected by upregulated FDX1 (Adrenal ferredoxin), ETFB (Electron Transfer Flavoprotein Subunit Beta), NDUFB4 (NADH: Ubiquinone Oxidoreductase Subunit B4) and NDUFA2 (NADH: Ubiquinone Oxidoreductase Subunit A2) (Figure 4B). Corresponding with gene expression, PaPE-1 treatment increased mitochondrial activity-related transporters and enzymes. VDAC1 (Voltage-dependent anion-selective channel 1), located in the outer mitochondrial membrane, allows ATP to diffuse out of the mitochondria into the cytoplasm. Cyt C (cytochrome complex), having an essential role in the electron transport chain, carries electrons from the bc1 complex to the complex IV. COX4 (cytochrome c oxidase subunit IV) is the last enzyme in the electron transport chain which drives oxidative phosphorylation. HSP60 (Heat shock protein) is a mitochondrial chaperonin that assists in the transport and proper refolding of certain proteins from the cytoplasm into the mitochondrial matrix. PHB (Prohibitin) in the inner membrane of mitochondria is essential for cell proliferation (Figure 4C) [18]. To understand mitochondrial metabolism pathways in the diet-induced obesity model, HepG2-ERα cells were treated with oleic acid (OA), PaPE-1, and OA + PaPE-1. Mitochondrial respiration parameters were measured using the Seahorse Mitostress kit. PaPE-1 treatment resulted in decreased proton leakage and increased coupling efficiency. 

### 3.4. PaPE-1 Decreased Collagen Deposition in Liver

**Figure 5:**
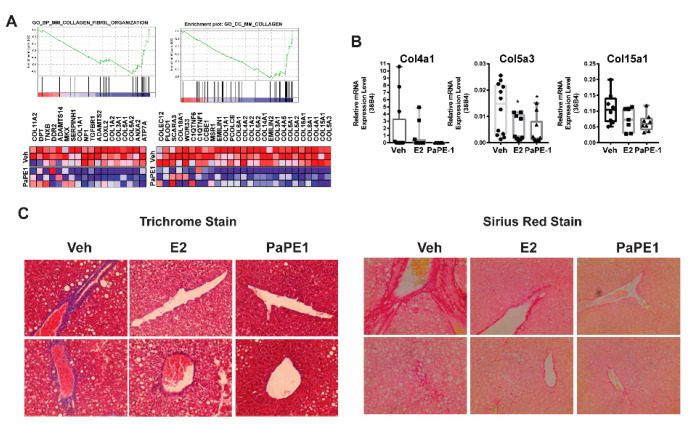
Decreased Collagen deposition in PaPE-1 treated mice livers (**A**) RNA-Seq analysis showed that PaPE-1 specifically downregulated collagen target genes. This is relevant to fibrosis, which is a hallmark of the next step after Nonalcoholic fatty liver disease (NAFLD), Nonalcoholic steatohepatitis (NASH). (**B**) PaPE-1 very effectively downregulated representative mice hepatic collagen genes in mice livers. (**C**) Trichrome and Sirius red staining showed PaPE-1 was very effective in decreasing mice’s hepatic collagen deposition (Blue in Trichrome stain, red in Sirius Red stain). *, *p* < 0.05.

Liver fibrosis, which is characterized by collagen deposition, is a common feature of chronic liver diseases, including NAFLD, and precedes liver cirrhosis. Staging fibrosis is essential in all patients with NAFLD to identify individuals with advanced fibrosis who are at risk of liver-related complications or Nonalcoholic steatohepatitis (NASH). In our DIO model, six weeks of feeding a high-fat diet led to detectable collagen deposition, and both E2 and PaPE-1 administration reduced hepatic collagen deposition significantly in both DIO mice. Gene expression analysis showed that PaPE-1 specifically downregulated highly enriched collagen target genes, which are essential biomarkers of fibrosis (Figure 5A). To validate these results in vivo, changes in the expression of representative collagen genes including COL4A1, COL5A3, and COL15A were assayed in liver samples from animals treated with Veh, E2, or PaPE-1 for six weeks. PaPE-1 more effectively modulated hepatic collagen genes compared with E2 (Figure 5B). Histological analysis of Trichrome and Sirius red staining provided evidence that PaPE-1 markedly ameliorated mouse hepatic collagen deposition (Figure 5C).

### 3.5. Metabolomics and Integrated Pathway Analysis Show Pape-1 Reduces Inflammation in the Liver

**Figure 6:**
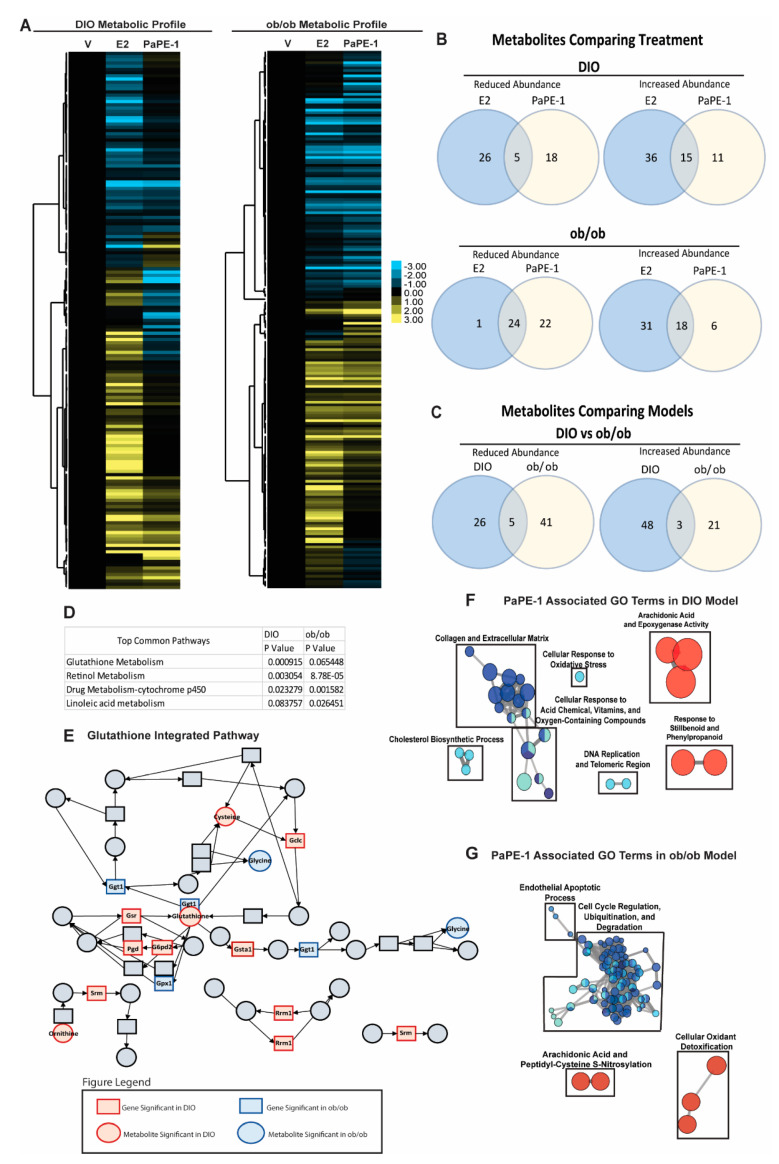
Metabolomics and integrated pathway analysis of PaPE-1 treatment in DIO and ob/ob mice. (**A**) Blood serum metabolites of PaPE-1 treatment in DIO and ob/ob mice. (**A**) Metabolites were identified by the GC-MS analysis and incorporated into a heatmap for visualization. Metabolites were clustered using Cluster 3.0 and visualized using Java TreeView. (**B**) PaPE-1 regulated several metabolites similarly to E2 treatment. (**C**) There were many metabolites distinct to each mouse model though a few were similar. (**D**) Integrated pathway analysis using Metaboanalyst showed common metabolic pathways affected by PaPE-1 treatment in both DIO and ob/ob models. The top common pathways between the two mouse models show retinol metabolism and drug metabolism-cytochrome p450 were significantly modulated. (**E**) The glutathione metabolism pathway is shown as an example of a significant pathway affected. Red denotes modulation in DIO mice and blue denotes modulation in ob/ob mice. GO term analysis of genes and metabolites of (**F**) DIO and (**G**) ob/ob models show several pathways affected by PaPE-1 treatment which show a possible reduction in inflammation and oxidative stress. Blue denotes downregulated GO terms and red denotes upregulated GO terms.

To understand changes in the composition of blood metabolites induced by various estrogens, we obtained plasma from animals at the time of harvest and performed a global metabolomics analysis. Blood plasma metabolites in DIO and ob/ob mice after estradiol or PaPE-1 treatment were identified using GC-MS analysis (Figure 6A). Based on this analysis, several metabolites were regulated similarly by PaPE-1 and E2, although many metabolites were differentially regulated (Figure 6B,C). Integrated pathway analysis was conducted using gene expression and metabolite composition data in Metaboanalyst 3.0 (Figure 6D). Several genes and metabolites correlated with the glutathione metabolism pathway in DIO and ob/ob mouse models, including Gsta1, which is significantly regulated in both DIO and ob/ob mouse models (Figure 6E). Integrated GO term analysis was performed using genes and metabolites significantly affected by PaPE-1 treatment. GO terms decreased by PaPE-1 treatment are highlighted in blue whereas increased GO terms are highlighted in red. In the DIO model, terms including cellular response to oxidative stress and cholesterol biosynthetic process were found to be decreased by PaPE-1 treatment. In the ob/ob model, terms including cell cycle regulation were decreased whereas cellular oxidant detoxification was increased (Figure 6F). Therefore, PaPE-1 targets certain inflammation and lipid metabolic pathways in DIO and ob/ob mouse models (Figure 6G). 

## 4. Discussion

Using transcriptomics, metabolomics, and liver histology analyses, we have characterized and compared the biological effects of a novel synthetic estrogen, PaPE-1, vs. estradiol on liver lipids, fibrosis, and inflammation in two mouse models of HFD-induced hepatosteatosis in ovariectomized female mice with low endogenous estrogens to mimic the postmenopausal condition. Replacement with these agents overall reduced weight gain and body fat and altered plasma metabolite composition and liver gene expression associated with lipid metabolism, mitochondrial activities, and collagen deposition. Genes regulating glutathione metabolism were also upregulated, while metabolites associated with this pathway were increased in the blood with PaPE-1 treatment. 

Metabolic syndrome is strongly associated with NAFLD and liver steatosis, and the prevalence of metabolic syndrome among women is increasing, although there is no such significant trend for men [19]. In our previous studies, PaPE-1 was shown to have a major impact on the development of fat depots in animals under low estradiol conditions, where PaPE-1 decreased the weight of all fat depots, including perigonadal, perirenal, and subcutaneous adipose tissues [12]. Notably, unlike E2, which has many of these effects, PaPE-1 does not increase the uterus weight in ovariectomized animals. Our current results extend upon these previous findings by showing additional liver benefits of PaPE1 in DIO and ob/ob mice. Estrogens have a protective effect against NAFLD and have been shown to reverse steatosis in both male and female animal models [7,20,21]. Lipid accumulation in the liver in the form of large cytosolic lipid droplets has been considered a factor in the “first hit” of the development of NAFLD [22]. Liver weights and hepatic lipid droplet size were decreased in both DIO and ob/ob mice, showing PaPE-1′s protective effects against the “first hit” in the development of NAFLD in both the genetic and diet-induced obesity models. We also observed an increased expression of mitochondrial genes and proteins, suggesting an increased energy consumption.

In addition to targeting factors in the “first hit” of NAFLD development, PaPE-1 modulated several metabolic pathways associated with the reduction of oxidative stress and fibrosis. Our RNASeq analysis showed that PaPE1 increased the expression of mitochondrial proteins and coupling efficiency. Together with increased glutathione synthesis pathways, these changes might result in efficient utilization of excess energy and mitigate excess reactive oxygen species production by active mitochondria, while preventing damage to hepatocytes. This observation might explain the decrease that we noted in collagen deposition that was more prevalent in PaPE-1 treated animals. Glutathione is a major antioxidant that prevents oxidative stress in the liver by quenching ROS. Human GSTs are a family of enzymes capable of detoxifying electrophilic molecules generated through oxidative stress [23]. Glutathione transferase (GST) isoforms and their mRNA levels, in particular, are increased with NAFLD progression [24]. Furthermore, GO terms associated with decreasing oxidative stress were enriched in both DIO and ob/ob models. In addition, we previously showed the metabolites in the same pathway to be upregulated by long-term broccoli feeding and correlated with improvement of liver health [25]. 

Though PaPE-1 affected similar metabolic pathways in both DIO and ob/ob mouse models, not all genes and metabolites were significantly regulated in a correlated manner. This could be due to the differences in gene expression and signaling in ob/ob mice. Previous studies have shown that although ob/ob mice develop steatosis, they are less prone to develop steatohepatitis and fibrosis compared to other mouse models, such as DIO mice [26,27,28]. These differences have been attributed to the characterization of leptin as an essential mediator of hepatic fibrosis [26,27]. This may also contribute to the differences in MRI fat mass seen between DIO and ob/ob mice. Overall, ob/ob mouse models are considered to more accurately depict a genetic model of obesity, which is often more difficult to ameliorate. 

## 5. Conclusions

Our results show that PaPE-1 elicited significant changes in inflammation and lipid metabolic pathways in two different mouse models that simulate metabolic dysfunction associated with loss of estrogen production at menopause. Based on the “two-hit hypothesis”, NAFLD is best treated by targeting both lipid accumulation and inflammation in the liver. It appears that PaPE-1 modulates pathways in both aspects of the “two-hit hypothesis”, suggesting that compounds of this class could have a protective effect against steatosis and NAFLD. Overall, our data suggest a beneficial role of PaPE-1 in protecting against liver steatosis and inflammation that can be caused by the loss of estrogens. In contrast to estradiol, PaPE-1 provides a metabolic benefit without stimulation of reproductive tissues such as the uterus. Therefore, PaPEs might provide a promising approach to avoid NAFLD progression in postmenopausal women with obesity and metabolic syndrome.

## Data Availability

RNASeq data will be deposited to GEO database and will be available to public upon acceptance of the manuscript.

## Supplementary Materials

The following are available online at https://www.mdpi.com/article/10.3390/nu13103334/s1.
