# Supplementary figures and images for: Pathway Preferential Estrogens Prevent Hepatosteatosis Due to Ovariectomy and High-Fat Diets
Source: Nutrients. 2021 Sep 23;13(10):3334. doi: 10.3390/nu13103334 (PMC8540756; doi:10.3390/nu13103334)

# PaPE-regulated pathways

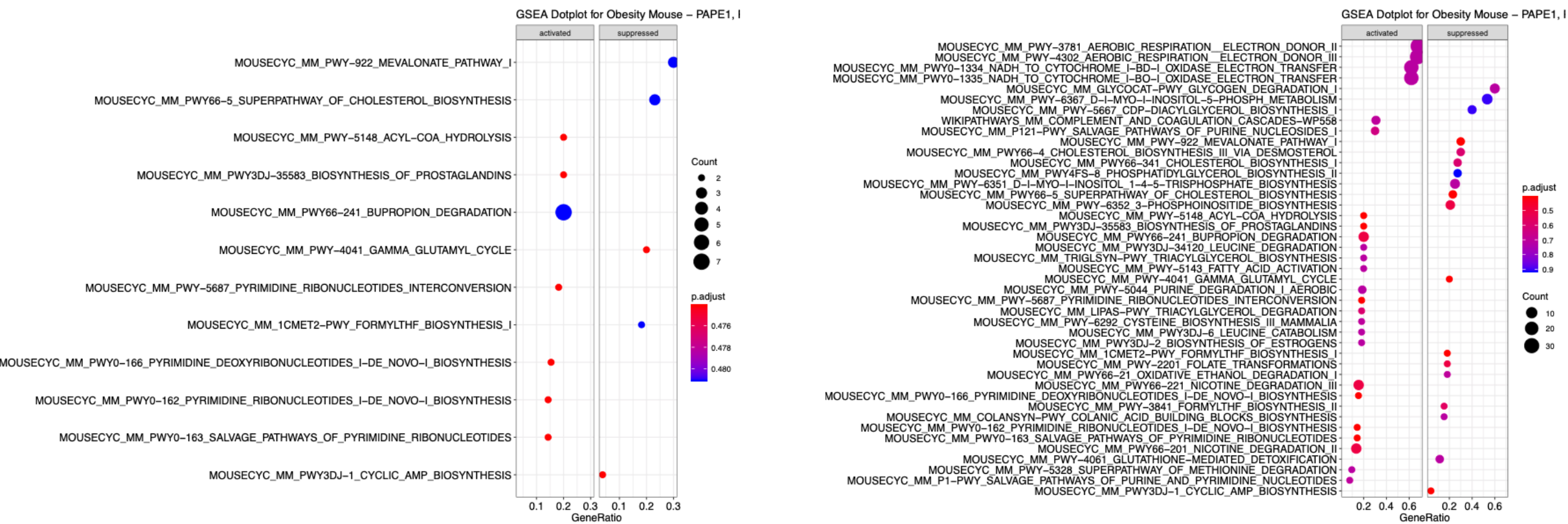

# E2-regulated pathways

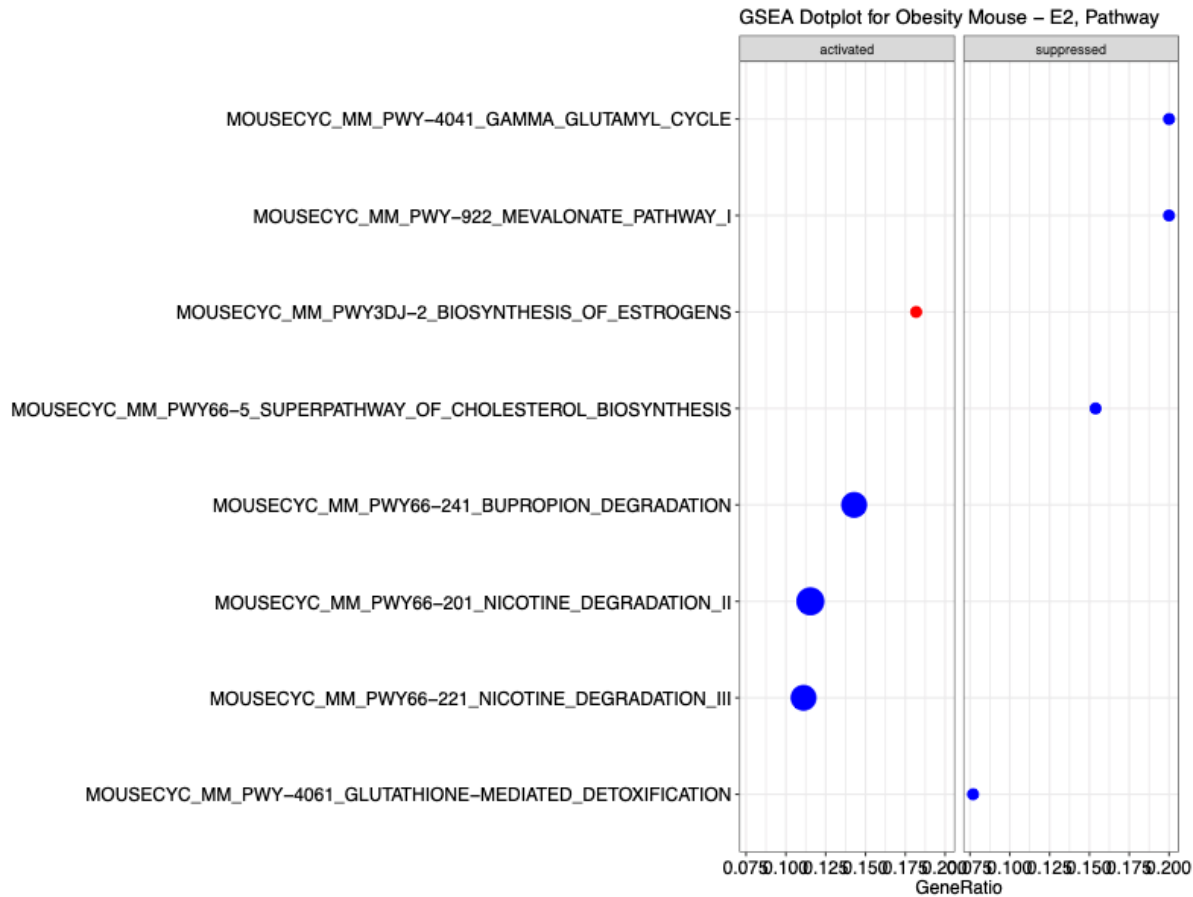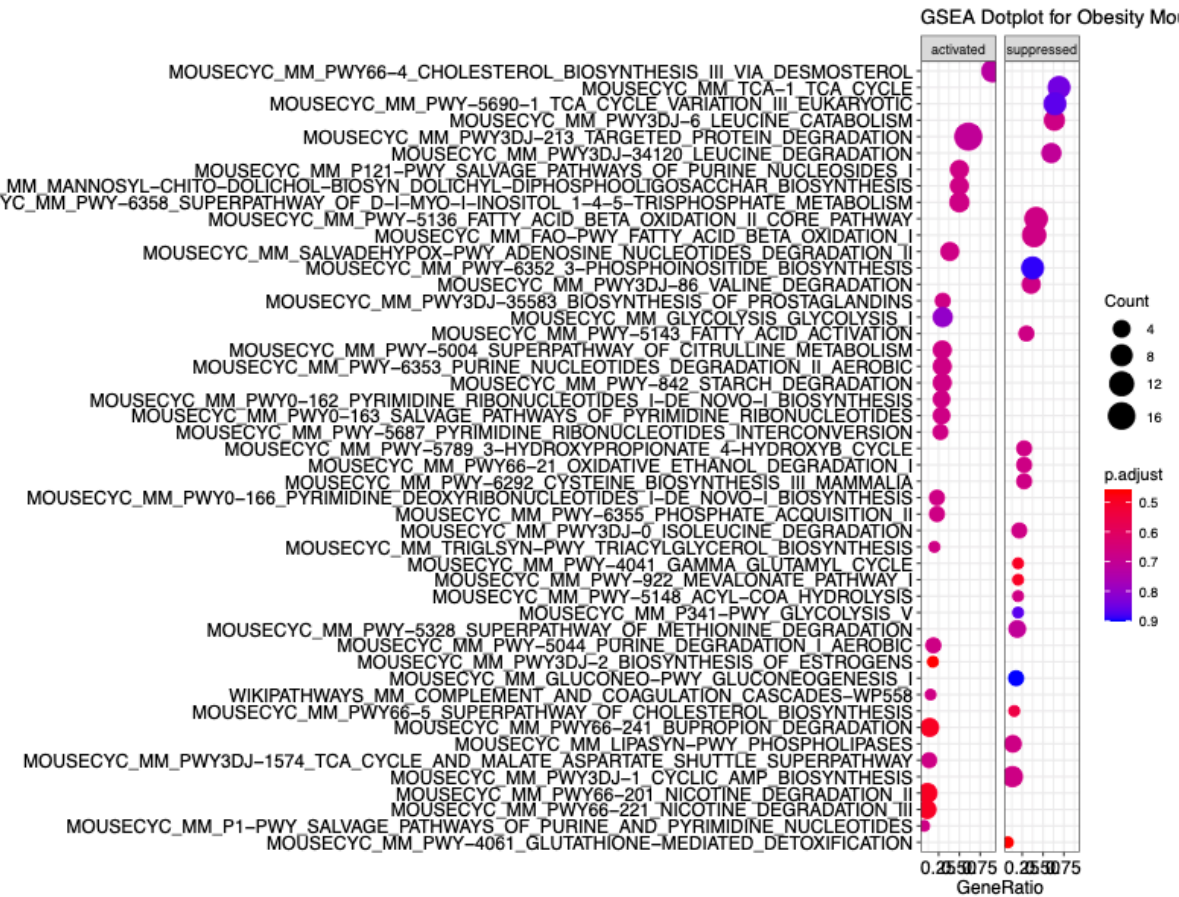

Supplement: Supplementary file 1 [file nutrients-13-03334-s001.zip › nutrients-1335619-supplementary.pdf]
